# Inflammation-linked adipokine profiles associate with acute organ dysfunction after severe trauma

**DOI:** 10.1186/s12967-026-08968-4

**Published:** 2026-09-10

**Authors:** Lena Schütte, Katharina Oßwald, Marco Mannes, Lisa Wohlgemuth, Enes Kumral, Benjamin Mayer, Jasmin M. Bülow, Christian B. Bergmann, Borna Relja, Florian Gebhard, Markus Huber-Lang, Pamela Fischer-Posovszky, Farahnaz Rayatdoost, Rebecca Halbgebauer

**Affiliations:** 1 Institute of Clinical and Experimental Trauma Immunology, Ulm University Medical Center, Ulm, Germany; 2 Institute of Epidemiology and Medical Biometry, Ulm University, Ulm, Germany; 3 Translational and Experimental Trauma Research, Department of Trauma-, Hand-, Plastic- and Reconstructive Surgery, University Ulm Medical Center, Ulm, Germany; 4 Department of Trauma-, Hand-, Plastic- and Reconstructive Surgery, University Ulm Medical Center, Ulm, Germany; 5 Department of Pediatrics and Adolescent Medicine, Ulm University Medical Center, Ulm, Germany; 6 German Center for Child and Adolescent Health (DZKJ), Partner Site Ulm, Ulm, Germany

**Keywords:** Adipokines, Multi-organ dysfunction syndrome, Sequential organ failure assessment score, Polytrauma

## Abstract

**Introduction:**

Polytrauma initiates an abrupt shift in immunometabolic signalling, but the role of circulating adipokines in acute post-traumatic organ dysfunction is not fully understood. Resistin and adiponectin are circulating immune-metabolic markers at the interface of inflammation, metabolic stress, and renal dysfunction, but their dynamic response to trauma and their clinical relevance remain uncertain. We therefore characterised serial changes in circulating adipokines during the first 10 days after severe multiple trauma and evaluated their associations with systemic inflammation, organ-specific dysfunction and clinical outcomes. We additionally explored associations between CT-derived waist-to-hip ratio (WHR), as a marker of body-fat distribution, and adipokine profiles.

**Methods:**

This study represents a secondary retrospective analysis of prospectively collected plasma samples and clinical data from a longitudinal observational cohort, including 67 patients with Injury Severity Score ≥ 16 and 31 healthy volunteers. Plasma resistin, adiponectin, leptin and retinol-binding protein 4 (RBP4) were measured at admission (D0) and on D1, D5 and D10. Longitudinal changes were analysed using linear mixed-effects models adjusted for age, sex, BMI and WHR. Associations with inflammatory markers and organ dysfunction were assessed using partial Spearman correlations. Exploratory receiver-operating characteristic (ROC) analyses evaluated adipokine-based classification of hospital length of stay (LOS), ICU LOS, shock severity and 30-day mortality.

**Results:**

Compared with healthy volunteers, trauma patients showed an altered adipokine profile at admission, characterised by elevated resistin (*p* < 0.01) and reduced adiponectin (*p* < 0.05). At admission, resistin correlated strongly with IL-6 (*r* ≈ 0.75), which remained significant after FDR correction. The resistin-to-adiponectin (R/A) ratio demonstrated the most consistent associations across inflammatory and renal parameters. Most WHR-stratified comparisons showed no clear separation across groups. In exploratory ROC analyses, several adipokine measures showed moderate classification of selected clinical outcomes; however, mortality analyses were limited by few available events at later timepoints.

**Conclusions:**

Severe trauma was associated with rapid changes in circulating adipokine profiles, characterised by early resistin elevation, lower circulating adiponectin, and an increased R/A ratio. Resistin and the R/A ratio showed the most consistent associations with systemic inflammation and renal dysfunction. WHR-stratified differences for most adipokines were limited. Clinical outcome associations were exploratory and require prospective multicentre validation to establish added value beyond conventional clinical parameters.

**Graphical Abstract:**

**Figure:**
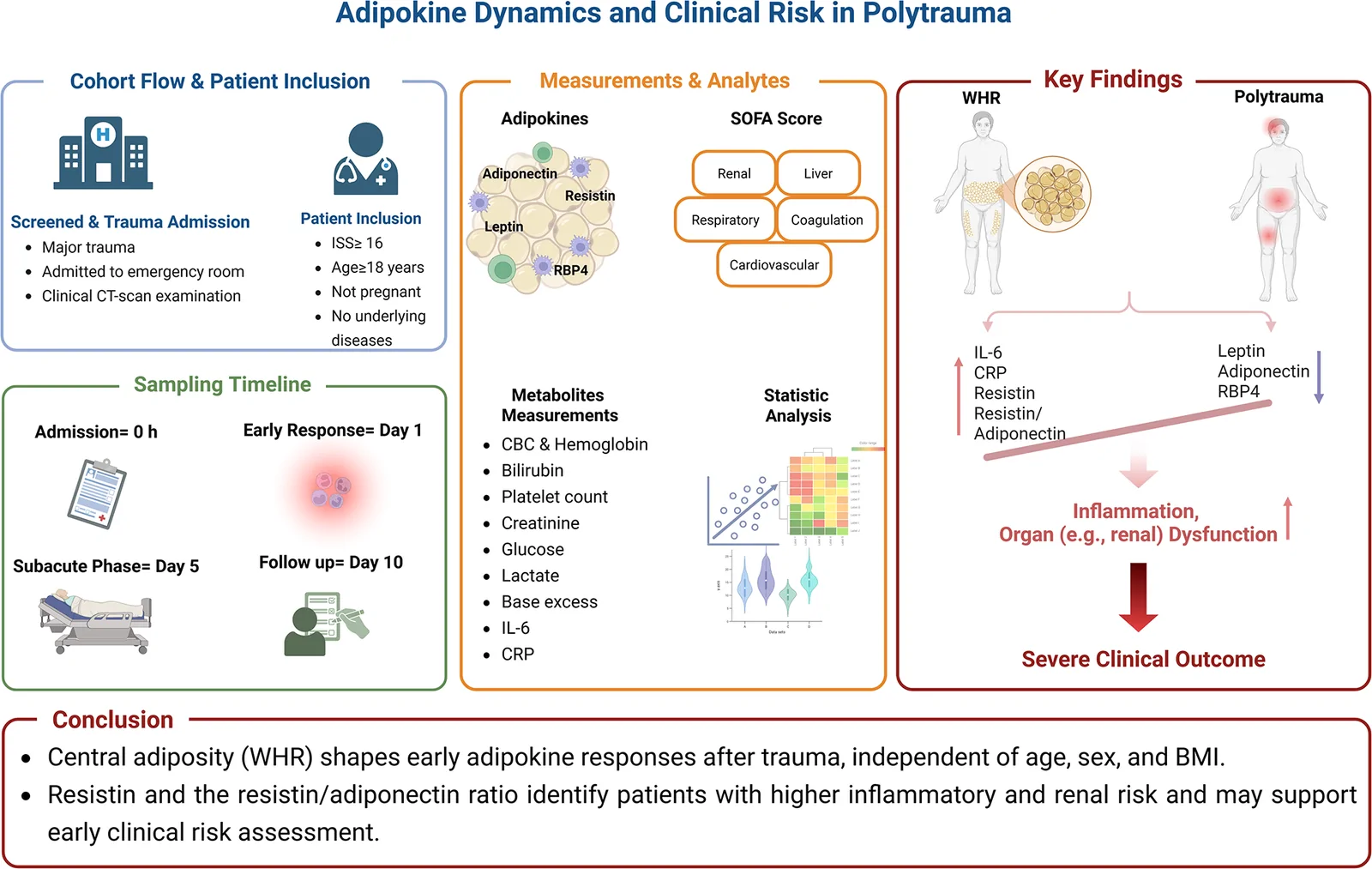

**Supplementary Information:**

The online version contains supplementary material available at https://doi.org/10.1186/s12967-026-08968-4.

## Introduction

Polytrauma (PT), defined by an Injury Severity Score (ISS) ≥ 16, poses a substantial risk for the development of multiple organ dysfunction syndrome (MODS), which remains a major driver of mortality in the intensive care unit (ICU) [1–3]. MODS arises from a complex interplay of systemic inflammation and immune dysregulation and is routinely assessed using the Sequential Organ Failure Assessment (SOFA) score to quantify organ failure and stratify mortality risk [2–4]. Despite advances in trauma care, early identification of patients at risk for MODS remains a clinical challenge.

White adipose tissue (WAT) has emerged as a biologically active endocrine and immunomodulatory organ, yet its role in the acute phase after trauma has received limited attention. WAT secretes numerous adipokines, including cytokines, chemokines, growth factors, and exosomal microRNAs, that influence systemic inflammation, glucose metabolism, endothelial activation, and inter-organ communication [5–8]. Dysregulation of adipokines is well described in metabolic disorders such as insulin resistance and type 2 diabetes mellitus [6, 9, 10].

Acute injury can perturb adipose-tissue metabolism and circulating adipokine concentrations; however, the measured markers have distinct cellular sources and should not be interpreted as a uniform adipose-tissue secretory response. In particular, human resistin is predominantly produced by monocytes and macrophages and is therefore more closely linked to myeloid and innate immune activation than to adipocyte secretion [11–14]. Similarly, circulating RBP4 is predominantly hepatocyte-derived in humans and therefore should not be interpreted as a specific marker of adipose-tissue secretion [15].

Understanding this broader immune-metabolic interplay is particularly relevant because a substantial proportion of trauma patients present with increased adiposity, altered metabolic profiles, and obesity-associated inflammation [8, 16–19]. Body fat distribution, particularly visceral adiposity, may further modulate the inflammatory response and susceptibility to organ dysfunction after severe injury [20–22]. However, previous studies investigating individual adipokines in critically ill or trauma patients have reported heterogeneous and sometimes conflicting results [16–18, 23, 24]. This highlights the need to define how acute trauma alters adipokine dynamics and whether these changes are associated with subsequent clinical outcomes.

Adipokine responses after severe trauma are likely shaped by the intensity of injury and shock, systemic inflammation, central fat distribution, sex, and metabolic factors such as catabolism and nutritional status. Resistin, adiponectin, leptin, and RBP4 therefore represent biologically heterogeneous circulating immune-metabolic and organ-stress signals.

Beyond single biomarkers, adipokine ratios such as leptin/adiponectin (L/A) and adiponectin/resistin (A/R) have been investigated in metabolic and cardiometabolic disorders as integrated markers of inflammatory and metabolic state [10, 25–32]. Whether such composite indices reflect early immunometabolic stress and organ dysfunction trajectories in severe polytrauma remains incompletely defined. We therefore analysed L/A and R/A ratios as exploratory composite indices alongside the individual adipokine trajectories.

In this study, we performed longitudinal profiling of resistin, adiponectin, leptin, and RBP4, and their ratios, over the first 10 days after severe trauma. Combining mixed-effects modelling, CT-based assessment of waist-to-hip ratio (WHR), partial correlation analysis, and organ-specific clinical endpoints, we aimed to characterise the early adipokine response, determine its relationship with inflammation and organ dysfunction, and perform exploratory analyses of the relationships between adipokine measures prespecified clinical outcomes in severely injured patients.

## Methods

### Study design and ethical approval

In this study, we performed a secondary retrospective analysis of prospectively collected samples and clinical data from a longitudinal observational study of severely injured polytrauma patients treated at a Level I Trauma Center, Ulm University Hospital, Ulm, Germany. The study was approved by the Ethics Committee of Ulm University (244/11, 94/14, and 260/22), and written informed consent was obtained from all participants or their legal representatives in accordance with the Declaration of Helsinki.

### Patient enrollment and clinical characterization

Adult patients (≥ 18 years) with severe multiple injuries, defined by an ISS ≥ 16 between 2013 and 2024 were included. Exclusion criteria comprised pregnancy, HIV infection, cardiogenic shock as primary diagnosis, hematologic malignancy, recent cytotoxic therapy, or terminal disease with expected survival < 24 h. Clinical evaluation followed standardized trauma protocols including whole-body CT imaging. Detailed inclusion/exclusion criteria are provided in Supplementary Methods.

### Sampling schedule and clinical laboratory measurements

Peripheral blood was collected at admission (D0), 24 h (D1), day 5 (D5), and day 10 (D10), representing the acute injury, early post-resuscitation inflammatory, subacute organ dysfunction, and early in-hospital follow-up phases, respectively. Complete sampling at all four timepoints was not required for study inclusion. Longitudinal mixed-effects models used all available repeated adipokine measurements among participants with complete data for the covariates included in the respective model; repeated outcome measurements were not imputed. Available adipokine measurements decreased over time because of death, discharge, or unavailable samples: D0 *n* = 66, D1 *n* = 60, D5 *n* = 58, and D10 *n* = 48. Samples were obtained under emergency and ICU conditions; fasting and nutritional status were not standardized or systematically available for adjustment. Detailed exposure data for nutritional support and medications relevant to adipokine biology, including catecholamines, corticosteroids, and insulin, as well as pre-existing metabolic conditions such as diabetes or metabolic syndrome, were unavailable or incompletely captured and therefore could not be included as covariates. Plasma processing and storage followed standard operating procedures (see Supplementary Methods). Routine laboratory parameters, including IL-6, CRP, lactate, glucose, base excess, bilirubin, platelet counts, and creatinine, were obtained from the hospital’s certified central laboratory.

### Assessment of circulating resistin, adiponectin, leptin, and RBP4

Plasma concentrations were measured using commercially available ELISAs (R&D Systems; for analytical ranges and assay details, see Supplementary Methods). RBP4 was retained within the predefined circulating marker panel but, given its predominantly hepatic origin in humans, was not interpreted as a direct adipose-derived signal. Adipokine concentrations were analysed as measured plasma concentrations and were not normalized to hematocrit or total protein. L/A and resistin/adiponectin (R/A) ratios were calculated at each timepoint.

### Clinical endpoints and organ dysfunction scoring

Organ dysfunction was assessed using the SOFA score reconstructed at each sampling timepoint. Organ-specific SOFA sub-scores (renal, cardiovascular, respiratory, hepatic, coagulation) were analysed separately. Details of SOFA reconstruction, including handling of missing data and Richmond Agitation-Sedation Scale to Glasgow Coma Scale (RASS-to-GCS) conversion, are provided in Supplementary Methods. Hemorrhagic shock (HS) was defined as base excess < -6 mmol/L, lactate ≥ 2.5 mmol/L, or transfusion > 2 units of packed red blood cells within the first 24 h following admission [33]. This variable was used for clinical characterization rather than as a mechanistic exposure defining the primary adipokine analyses.

### Anthropometric assessment

WHR was measured from admission CT scans, using measurements at the level of the umbilicus for waist circumference and the femoral neck for hip circumference. WHR was used as a CT-derived proxy of central body-fat distribution. WHR categories were defined according to World Health Organization (WHO) criteria [34] and were used for descriptive stratification and visualization. Because these thresholds were developed for cardiometabolic risk assessment and are not validated trauma-specific cutoffs, WHR-stratified analyses were interpreted descriptively. Patients were stratified into three WHR categories. For women, moderate WHR was defined as 0.85 ≤ WHR < 0.95. For men, moderate WHR was defined as 0.90 ≤ WHR < 1.00. Values below these ranges were classified as low WHR, and values equal to or above these ranges as high WHR.

### Statistical analysis

Statistical analyses and visualisations were performed in R, with additional figures in GraphPad Prism 9.1.2. Continuous variables are presented as mean ± SD or median [IQR] as appropriate. Admission group comparisons were conducted using multivariable linear regression adjusted for age, sex, and BMI.

Longitudinal adipokine changes were analysed using linear mixed-effects models with a random intercept for patient ID and fixed effects for time, age, sex, BMI, and WHR, with WHR entered as a continuous covariate; log-transformation was applied where required, with model assumptions verified by residual and Q–Q plots. Post-hoc contrasts were adjusted using the Benjamini–Hochberg false discovery rate (FDR) within each biomarker-specific contrast set. For partial Spearman correlations, FDR correction was applied separately within two predefined correlation families: adipokines/adipokine ratios with inflammatory-metabolic and renal laboratory markers, and adipokines/adipokine ratios with clinical severity and SOFA-domain scores. Coefficients with |r| < 0.30 were considered weak and were not interpreted as biologically or clinically relevant, even when nominally significant.

As sensitivity analyses, partial Spearman correlations between admission (D0) adipokines/adipokine ratios and AIS regional scores were adjusted for age, sex, BMI, and global ISS, with FDR correction across all 36 region–marker comparisons. Selected D0 correlations were also repeated with and without additional adjustment for admission hematocrit to assess potential confounding by resuscitation-related hemodilution. ROC analyses were performed exploratorily for prespecified dichotomized clinical outcomes: shock ratio (> 1), hospital LOS (> 21 days), ICU LOS (> 7 days), and 30-day mortality. For clinical context, routinely available admission variables including ISS, shock ratio, base excess, lactate, IL-6, SOFA, creatinine, GFR, and CRP were evaluated using the same exploratory framework for hospital LOS > 21 days and 30-day mortality. These analyses were descriptive and were not intended as formal head-to-head comparisons of biomarker superiority. AUCs with 95% confidence intervals were estimated using the DeLong method. To assess whether R/A provided greater discrimination than resistin alone, paired AUCs were compared using DeLong tests among participants with both measurements available at the corresponding timepoint. Differences in AUC (ΔAUC = AUC_R/A_− AUC_Resistin_) are reported with 95% confidence intervals (Supplementary Fig. 2A). Reported p-values were derived from Mann–Whitney U tests comparing biomarker distributions between outcome groups and do not test whether an AUC differs from 0.5. No internal or external validation was performed, and the selected cutoffs were not intended as validated prognostic thresholds.

Analyses were based on available observations with complete data for the variables and covariates required by each respective analysis; missing data were not imputed. Linear mixed-effects models therefore included all available repeated measurements meeting these requirements. Their interpretation under incomplete follow-up relies on a missing-at-random assumption conditional on the observed data and covariates included in the model. Because later samples could be unavailable because of death, discharge, or clinical instability, this assumption cannot be verified in the present cohort. Accordingly, the mixed-effects models were used to describe the observed longitudinal patterns rather than to estimate population-wide D5–D10 trajectories. Potential attrition-related selection was characterised descriptively by comparing patients with and without an available D10 adipokine measurement; no inferential testing was performed for this comparison.

No formal a priori sample-size calculation was performed for this secondary exploratory analysis. WHR-stratified analyses, WHR × sex interaction models, and ROC analyses were therefore considered exploratory. All tests were two-sided (α = 0.05).

## Results

Sixty-seven polytrauma patients and 31 healthy volunteers (HV) were enrolled. Baseline characteristics are shown in Table 1. The trauma cohort had a mean age of 50 years and was predominantly male (78%). Injury severity was high, with a median ISS of 27 (IQR 21–33) and an admission shock ratio of 1.1 (IQR 0.9–1.4). Nine patients died within the first 10 days after trauma; in total, 14 patients died within 30 days.

**Table 1:** Cohort overview and key admission characteristics of healthy volunteers (HV) and trauma patients (PT)

Variable	HV (*n* = 31)	PT (*n* = 67)
**Demographics**		
Age (years), [range]	37 ± 14 [21–81]	50^*^ ± 18 [20–90]
Female sex, n (%)	9 (29.0%)	15 (22.3%)
Male sex, n (%)	22 (71.0%)	52 (77.6%)
WHR	—	0.94 ± 0.07 (0.82–1.11)
BMI (kg/m²) ^†^, mean ± SD	24.3 ± 3.1	26.2^*^ ± 3.2 (20–35)
**Laboratory at 0 h**,	***Lab ref range***	***median [IQR]***
Platelet count (×10⁹/L)	—	196.4 ± 60.2
Glucose (mg/dl)	74–109	128 [105–160]
Lactate (mmol/L)	0.5–2.2	2.1 [1.2–3.6]
GFR (ml /min)		85 [68–102]
IL-6 (pg/ml)	< 7	220 [100–560]
CRP (mg/L)	< 5	0.9 [0.6–2.5]
Creatinine (µmol/L)	Female: 45–84 Male: 59–104	87 [70–112]
**Injury severity / physiology**		***median [IQR]***
ISS	—	27 [21–33]
Shock, n (%)	—	25/45 (55.6%)
Shock ratio	—	1.1 [0.9–1.4]
Base excess (mmol/L)	—	-2.4 [-4.2–-0.2]
**Clinical Outcomes**		***median [IQR]***
ICU length of stay (days)	—	5.5 [2–17]
Hospital length of stay (days)	—	21 [14–34.5]
30-day mortality, n (%)	—	14 (20.9%)

Values are presented as mean ± SD for normally distributed variables and median [IQR] for skewed variables. Differences between HV and PT for Age and BMI were tested using Welch’s t-test (*p < 0.05). BMI= Body Mass Index

### Temporal dynamics of adipokine levels after polytrauma

Complete serial sampling was not required; the number of patients with at least one available adipokine measurement decreased from D0 (*n* = 66) to D1 (*n* = 60), D5 (*n* = 58), and D10 (*n* = 48). Accordingly, later timepoint estimates describe patients with samples available at those timepoints rather than the complete admission cohort. To characterize potential attrition-related selection, baseline characteristics were compared descriptively between patients contributing at least one D10 adipokine measurement (*n* = 48) and those without a D10 adipokine measurement (*n* = 19; Supplementary Table 1). The comparison showed no uniform pattern of greater baseline severity in either group: median admission SOFA was 6 [4–8] in both groups, and admission resistin and adiponectin concentrations were similar descriptively, whereas D10 contributors had a higher median admission shock ratio and lower GFR. These descriptive findings do not establish that missingness was random.

**Fig. 1:**
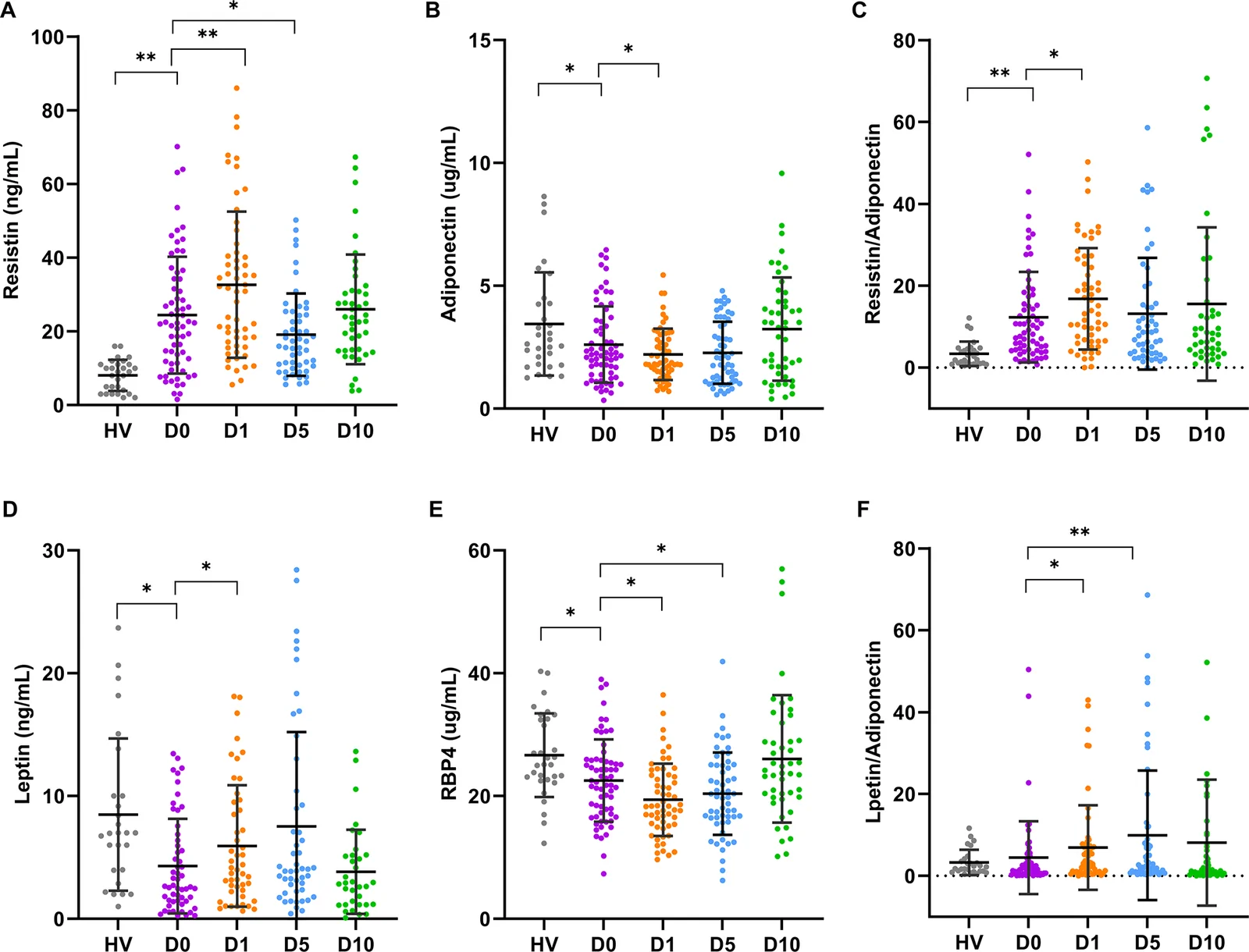
Changes in plasma adipokine levels over time after polytrauma. (**A**) Resistin, (**B**) Adiponectin, (**C**) Resistin/Adiponectin ratio, (**D**) Leptin, (**E**) RBP4, and (**F**) Leptin/Adiponectin ratio at admission (D0), 24 h (D1), day 5 (D5), and day 10 (D10), shown alongside healthy volunteer (HV) reference values. Trauma-patient changes over time were analysed using a linear mixed-effects model with fixed effects for time, age, sex, BMI, and WHR and a random intercept for patient ID. D0 comparisons between HV and PT were assessed using multivariable linear regression adjusted for age, sex, and BMI. Error bars represent mean ± SD. *p < 0.05, **p < 0.01, ***p < 0.001; FDR-corrected for repeated contrasts within PT. Available adipokine measurements were: healthy volunteers; *n* = 30, patients; D0, *n* = 66, D1, *n* = 60; D5, *n* = 58; D10, *n* = 48

Adipokine levels changed rapidly after injury (Fig. 1). Resistin was elevated at admission compared with HV (*p* < 0.01), increased further to a peak on D1 (*p* < 0.01 vs. D0), and declined thereafter. In contrast, adiponectin was already reduced at admission (*p* < 0.05), reached its lowest level on D1, and increased gradually thereafter. Together, these opposite directional changes produced an early rise in the R/A ratio, most pronounced within the first 24 h (*p* < 0.01).

Leptin and RBP4 were lower at admission than in HV (both *p* < 0.05). Leptin increased modestly on D1, whereas RBP4 decreased further from D0 to D1 (*p* < 0.05) and then returned toward D0 levels by D10. The L/A ratio was elevated at D0 and D1.

### CT-derived WHR and exploratory sex-related adipokine patterns

**Fig. 2:**
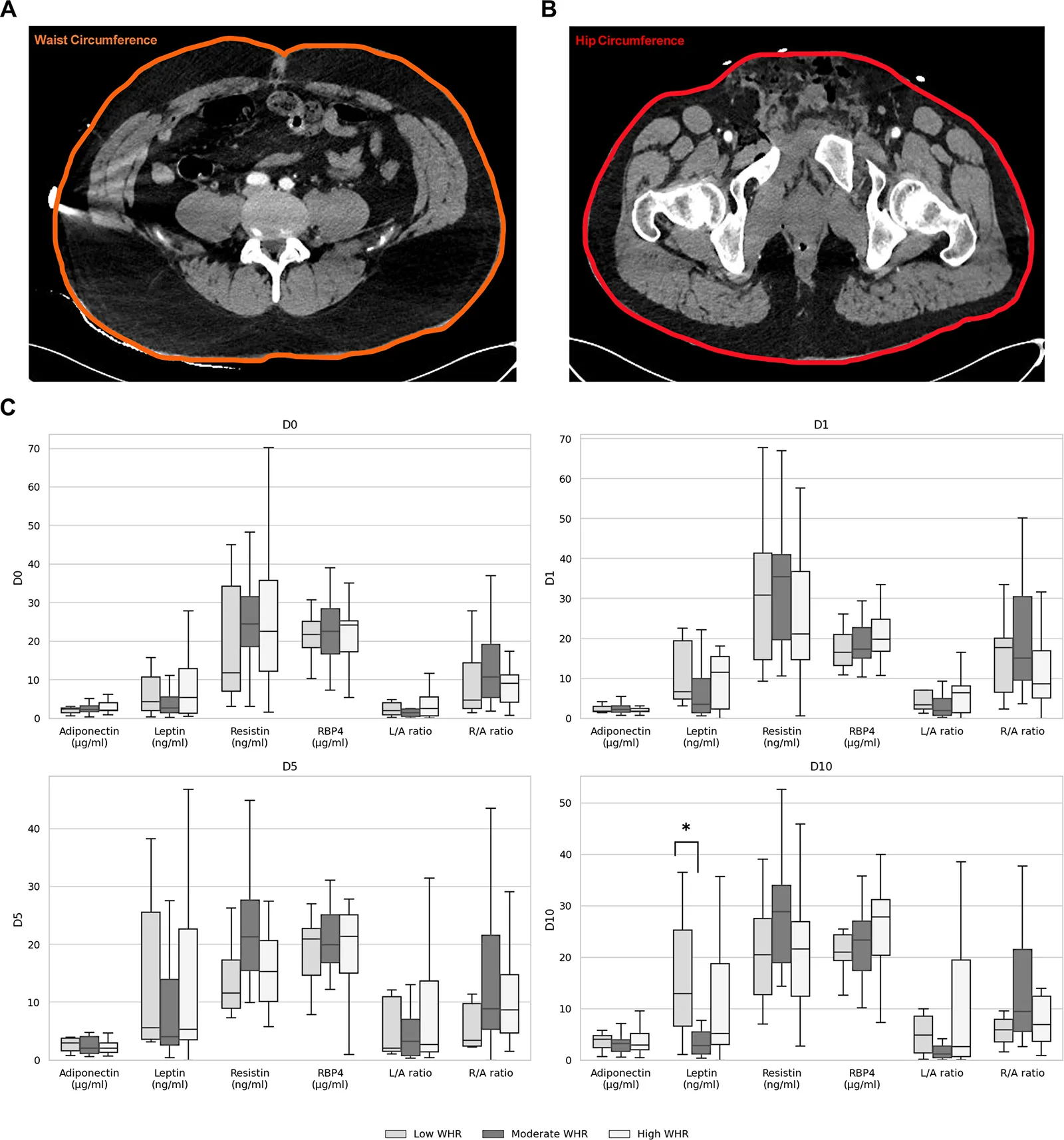
CT-derived WHR measurement and adipokine concentrations across WHR groups. (**A**–**B**) Axial CT images demonstrating the measurement of waist circumference (**A**) and hip circumference (**B**) used to calculate the waist-to-hip ratio (WHR). Measurement planes were standardised across patients. (**C**) Adipokine concentrations (adiponectin (ug/ml), leptin (ng/ml), resistin (ng/ml), RBP4 (ug/ml)) and adipokine ratios (Leptin/Adiponectin, Resistin/Adiponectin) at D0, D1, D5 and D10, stratified by WHR group (low, moderate, high). Boxes represent median and IQR; whiskers indicate full range. Statistical comparisons were performed using linear models adjusted for age, sex and BMI. Significant differences shown as *p < 0.05. Available adipokine measurements were: D0, HV; *n* = 30, PT; *n* = 66, D1, *n* = 60; D5, *n* = 58; D10, *n* = 48

WHR was measured from admission CT scans using axial measurements at the level of the umbilicus (waist) and femoral neck (hip) (Fig. 2A-B). Across all time points, adiponectin tended to be lower in high-WHR patients, but WHR-stratified differences were not statistically significant (Fig. 2C). By D10, leptin concentrations were higher in patients with moderate WHR (*p* < 0.05). Overall, WHR-stratified differences were limited. Therefore, WHR-related findings were interpreted mainly as exploratory patterns rather than definitive subgroup effects.

Exploratory WHR × sex interaction analyses showed different WHR–adiponectin slopes between women and men at D1 and D5 (Supplementary Fig. 1). Interaction terms reached nominal statistical significance at both timepoints (*p* < 0.05). However, only 15 women were included in the cohort, and the interaction estimates had wide confidence intervals. These analyses were therefore considered hypothesis-generating and were not interpreted as evidence of a definitive sex-specific effect. No comparable interaction pattern was observed for leptin, resistin, RBP4, or the adipokine ratios.

### Adipokine associations with inflammation and renal dysfunction

**Fig. 3:**
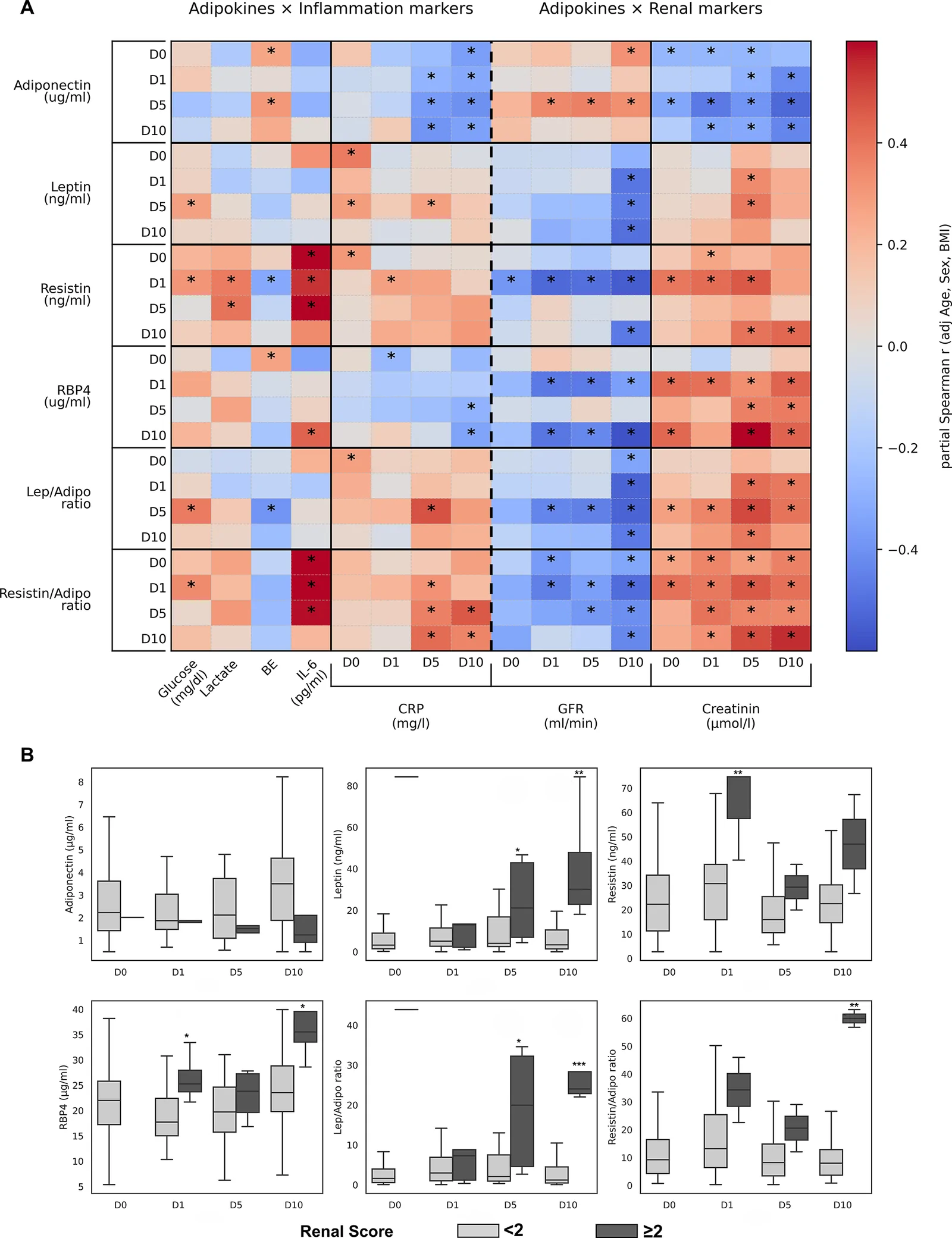
Associations between adipokines, inflammation, and renal function. (**A**) Heatmap showing partial Spearman correlation coefficients between adipokines (adiponectin, leptin, resistin, RBP4, L/A ratio, R/A ratio) and inflammatory-metabolic markers (IL-6, CRP, lactate, glucose) and renal labratory markers (creatinine, GFR) across D0, D1, D5 and D10. Correlations were adjusted for age, sex and BMI. * Asterisks indicate nominal statistical significance. Correlations were interpreted in the text only when they remained significant after Benjamini–Hochberg FDR correction. (**B**) Adipokine concentrations at each timepoint stratified by renal SOFA category (< 2 vs. ≥ 2). Boxes represent median and IQR; whiskers indicate full range. Comparisons were performed using linear models adjusted for age, sex and BMI. Statistical significance after adjustment shown as *p < 0.05, **p < 0.01, ***p < 0.001. *For renal SOFA-stratified analyses*,* patients were included at each timepoint when both the renal SOFA category and the corresponding adipokine measurement were available. No additional clinical exclusions were applied. At D0*,* one patient met criteria for renal SOFA score 4; resistin and RBP4 measurements were unavailable and excluded from the corresponding analyses*

Resistin showed the strongest associations with inflammatory markers. At admission, resistin correlated strongly with IL-6 (*r* = 0.75) and remained significant after FDR correction (Fig. 3A).

Among renal laboratory markers, the strongest association patterns involved resistin and the R/A ratio. D1 resistin correlated inversely with GFR at D1 (*r*=-0.52), D5 (*r*=-0.50), and D10 (*r*=-0.55); all three associations remained significant after FDR correction. D1 resistin correlated positively with creatinine at D1 (*r* = 0.42) and D5 (*r* = 0.46), both remaining significant after FDR correction. The R/A ratio showed a similar renal pattern. D1 R/A was associated with D0 and D5 creatinine (*r* = 0.41 and 0.46, respectively) and inversely with D10 GFR (*r*=-0.51), while D10 R/A correlated with D10 creatinine (*r* = 0.55); these associations remained significant after FDR correction. RBP4 showed additional late renal associations, with inverse correlation to GFR at D10 (*r* = -0.58) and positive correlation with creatinine at D5 (*r* = 0.63); both associations remained significant after FDR correction. Leptin showed fewer and less consistent associations than resistin or the R/A ratio (Fig. 3A). Overall, resistin and the R/A ratio showed the most consistent FDR-supported associations with inflammatory and renal laboratory markers (Fig. 3A). In adjusted comparisons, patients with renal SOFA ≥ 2 had significantly higher resistin at D5 and D10 (both *p* < 0.01), higher R/A ratios at both timepoints (*p* < 0.01), and higher RBP4 at D10 (*p* < 0.05). Adiponectin did not differ significantly by renal SOFA category. Full correlation coefficients are provided in Supplementary Table 2.

In an exploratory admission sensitivity analysis, additional adjustment for hematocrit produced little change in the strongest D0 associations, particularly resistin–IL-6 and R/A–IL-6, whereas several weaker correlations were attenuated or shifted (Supplementary Fig. 2B); thus, this analysis could not confirm whether absolute adipokine concentrations were influenced by hemodilution.

### Adipokine associations with shock severity and organ dysfunction

Resistin at D1 showed the strongest association with admission shock ratio (*r* ≈ 0.62), which remained significant after FDR correction. D1 R/A showed a similar adjusted association with admission shock ratio (*r* ≈ 0.55), also remaining significant after FDR correction.

**Fig. 4:**
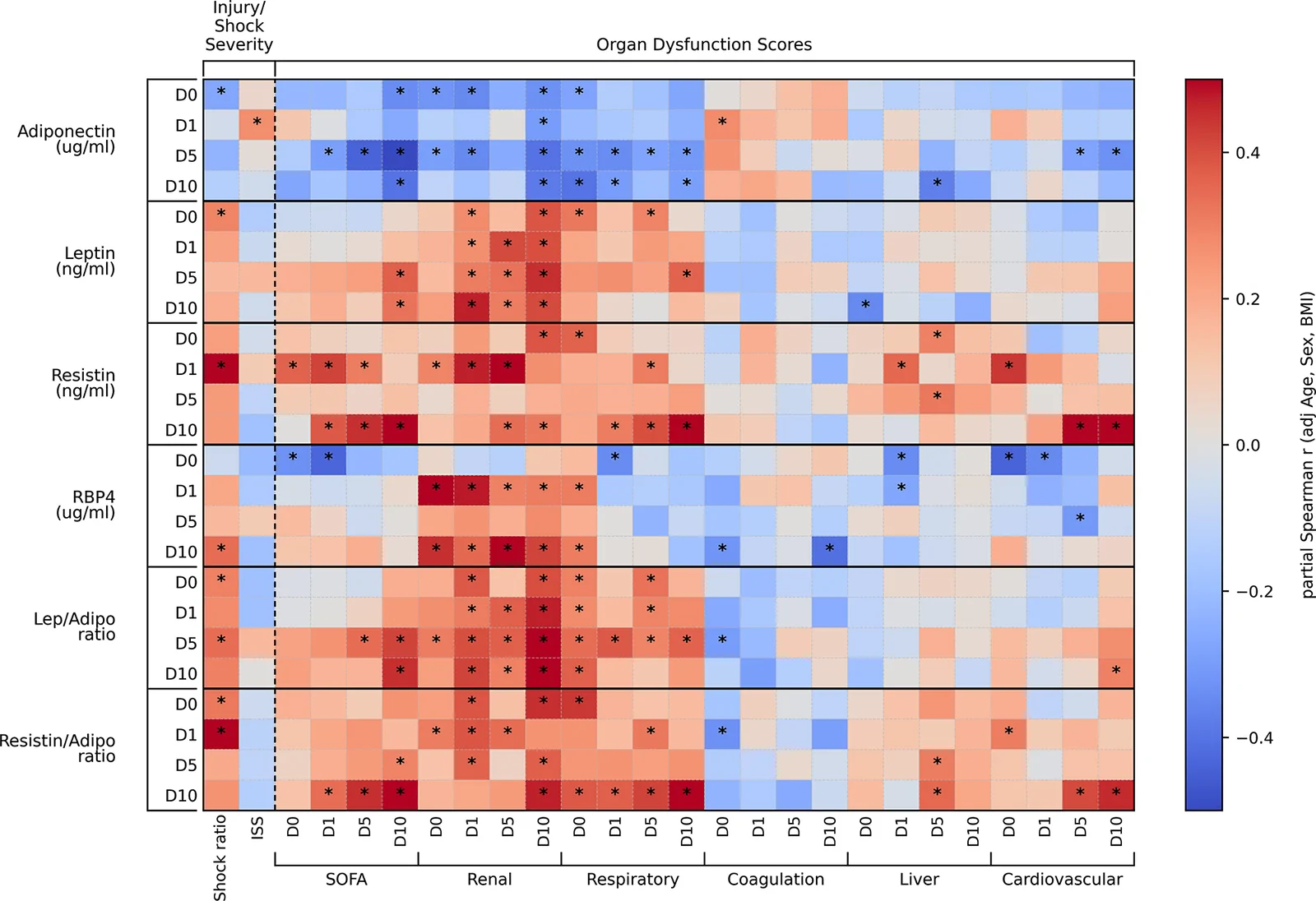
Adjusted correlations between adipokines, shock severity, and organ dysfunction scores. Heatmap displaying partial Spearman correlation adjusted for age, sex, and BMI between adipokines (adiponectin, leptin, resistin, RBP4, Leptin/Adiponectin ratio, Resistin/Adiponectin ratio) and clinical severity indices. Variables include admission shock ratio and SOFA-derived organ dysfunction domains (global SOFA, renal, respiratory, coagulation, hepatic, cardiovascular) at D0, D1, D5 and D10

FDR-supported associations with global SOFA were most evident at later timepoints. D10 resistin and D10 R/A correlated positively with D10 global SOFA (*r* = 0.57 and *r* = 0.60, respectively), whereas D5 adiponectin correlated inversely with global SOFA at D5 (*r*=-0.44) and D10 (*r*=-0.50) (Fig. 4).

Distinct organ-specific patterns emerged. Among the organ-specific SOFA domains, renal dysfunction showed prominent early associations. D1 resistin correlated with renal SOFA at D1 (*r* = 0.47) and D5 (*r* = 0.53), with both associations remaining significant after FDR correction. These correlations indicated close alignment between the early inflammatory adipokine profile and renal dysfunction but did not establish temporal directionality or causality. Late-phase cardiovascular and respiratory dysfunction also showed adipokine associations: D10 resistin correlated strongly with cardiovascular SOFA (*r* = 0.51) and respiratory SOFA (*r* = 0.58), with both associations remaining significant after FDR correction. Comprehensive adjusted coefficients for all timepoints are reported in Supplementary Table 2. In exploratory baseline-adjusted lagged analyses, none of the 18 associations between D0 resistin, adiponectin, or R/A and subsequent D1 or D5 renal SOFA, creatinine, or GFR remained significant after FDR correction (all q > 0.05; Supplementary Table 4B). Thus, the present data did not demonstrate that admission adipokine levels preceded subsequent renal dysfunction independently of renal status at admission.

### Exploratory ROC analyses of adipokines and clinical outcomes

Due to relatively limited patient numbers, ROC analyses were performed in an exploratory fashion and based on prespecified dichotomized clinical outcomes; therefore, the resulting AUCs pertain to these specific outcome definitions and were not used to derive validated clinical thresholds. Reported p-values are descriptive Mann–Whitney U tests comparing biomarker distributions between outcome groups and are not tests of whether an AUC differs from 0.5. Most AUCs were in the fair-to-moderate range, and nominal p-values should be interpreted cautiously in view of multiple testing and limited event numbers. For ICU LOS > 7 days, leptin at D5 yielded an AUC of 0.72 (95% CI 0.53–0.87), with a corresponding Mann–Whitney p-value of 0.014 (Supplementary Table 5A). For hospital LOS > 21 days, the L/A ratio at D5 yielded an AUC of 0.70 (95% CI 0.54–0.85), with a corresponding Mann–Whitney p-value of 0.016. R/A yielded AUCs of 0.67 at D1 and 0.71 at D10, with corresponding Mann–Whitney p-values of 0.041 and 0.028, respectively (Supplementary Table 5B).

For admission shock ratio, L/A and R/A yielded AUCs of 0.69 and 0.66, respectively, with corresponding Mann–Whitney p-values of 0.012 and 0.030. Admission adiponectin was inversely associated with shock classification (AUC 0.35; *p* = 0.039) (Supplementary Table 5B).

**Fig. 5:**
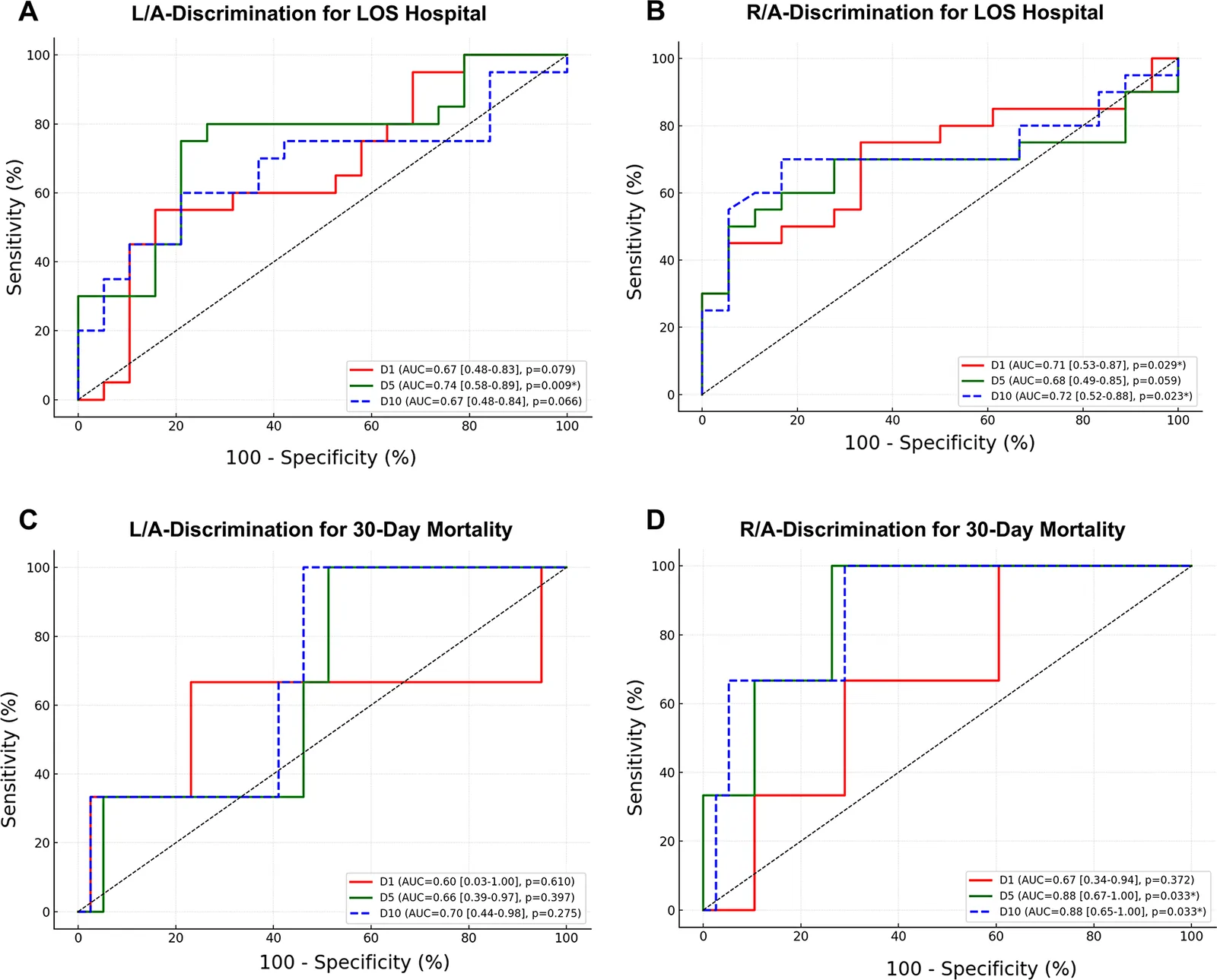
Exploratory ROC analyses of adipokine ratios for hospital length of stay and 30-day mortality. Curves show L/A for hospital LOS > 21 days (**A**) and mortality (**C**), and R/A for hospital LOS > 21 days (**B**) and mortality (**D**). For serial comparability, each panel uses participants with complete ratio measurements at D1, D5, and D10: L/A LOS *n* = 39 (21 events), R/A LOS *n* = 38 (20 events), L/A mortality *n* = 42 (3 deaths), and R/A mortality *n* = 41 (3 deaths). AUC 95% confidence intervals were estimated by DeLong’s method. Displayed p-values are two-sided Mann-Whitney U tests of biomarker distributions between groups, not tests comparing AUCs. The mortality estimates are highly imprecise and exploratory; no validated prediction threshold was derived

Mortality ROC analyses were particularly limited by the small number of events represented at later sampling timepoints (Fig. 5). Although 14 patients died within 30 days in the overall cohort, the available R/A datasets included 51 patients with 7 deaths at D1, 47 patients with 4 deaths at D5, and 42 patients with 3 deaths at D10. The corresponding R/A AUCs were 0.71 (95% CI 0.52–0.89) at D1, 0.88 (95% CI 0.72–1.00) at D5, and 0.86 (95% CI 0.63–1.00) at D10 (Fig. 5). Given the very small numbers of deaths, particularly at D5 and D10, these estimates are highly uncertain and are reported solely as exploratory, hypothesis-generating observations rather than evidence of prognostic performance.

Paired DeLong comparisons were used to examine whether R/A provided greater discrimination than resistin alone in the same participants (Supplementary Fig. 2A). For hospital LOS > 21 days, the D0 comparison favored R/A (ΔAUC 0.13, 95% CI 0.01–0.26), whereas the confidence intervals for the D1, D5, and D10 comparisons included zero. For 30-day mortality, confidence intervals included zero at all four timepoints. Thus, the analyses did not demonstrate a consistent improvement in discrimination with R/A over resistin alone.

For clinical context, exploratory ROC analyses were also performed for routinely available admission variables (Supplementary Table 5C). For hospital LOS > 21 days, admission SOFA showed an AUC of 0.79 (95% CI 0.67–0.91), shock ratio 0.74 (95% CI 0.61–0.88), and IL-6 0.71 (95% CI 0.50–0.93). For 30-day mortality, admission SOFA showed an AUC of 0.70 (95% CI 0.54–0.86), with ISS and lactate yielding AUCs of 0.68 and 0.66, respectively. Thus, several routinely available clinical variables showed discrimination comparable to or higher than adipokine-based measures for selected outcomes. These descriptive analyses were not formal paired comparisons and do not establish relative superiority of either biomarker class.

## Discussion

Severe trauma was associated with rapid changes in circulating immune-metabolic markers. The dominant pattern was an early increase in resistin accompanied by lower circulating adiponectin, resulting in an increased R/A ratio. Among the measured markers, resistin and R/A showed the most consistent associations with systemic inflammation and organ dysfunction, particularly across renal laboratory measures and renal SOFA.

Human resistin is predominantly produced by monocytes and macrophages rather than adipocytes [11, 12], making its post-traumatic increase compatible with systemic myeloid and innate immune activation [14], although its cellular source was not assessed here. Resistin correlated strongly with IL-6 at admission and was positively associated with creatinine, inversely associated with GFR, and higher in patients with renal dysfunction at later timepoints, paralleling previous reports linking resistin with inflammation and organ dysfunction in critical illness [35, 36]. However, baseline-adjusted lagged analyses showed no FDR-significant associations between admission resistin, adiponectin, or R/A and subsequent D1 or D5 renal outcomes. Thus, these data support concurrent associations but not temporal precedence of adipokine changes over renal dysfunction.

The R/A ratio reflected the opposing trajectories of increased resistin and reduced adiponectin and showed consistent associations with inflammatory and renal parameters. Similar adiponectin-based ratios have been studied as integrated markers of inflammatory and metabolic state in metabolic and cardiometabolic disorders [25–32]. In our cohort, however, R/A did not consistently outperform resistin alone: only the D0 comparison for hospital LOS > 21 days favoured R/A, while the remaining paired DeLong comparisons were compatible with no difference. In descriptive contextual analyses, several established clinical measures, including admission SOFA and shock ratio, also showed comparable or higher AUCs for selected outcomes. R/A should therefore be considered a descriptive composite rather than a marker with established incremental or superior prognostic value. The high mortality AUCs at D5 and D10, based on only four and three deaths, respectively, remain hypothesis-generating.

RBP4 and leptin showed patterns distinct from resistin. RBP4 was lower at admission than in healthy volunteers, decreased further by D1, and returned toward D0 levels thereafter, consistent with reduced circulating RBP4 reported in critical illness [36, 37]. Because circulating RBP4 is predominantly hepatocyte-derived, these changes should not be interpreted as reduced adipose-tissue secretion [15]. Potential contributors to the early decrease include hepatic hypoperfusion or ischaemia [38], negative acute-phase regulation [36], and resuscitation-related dilution, whereas altered renal handling may contribute to later renal associations [36]; these mechanisms were not directly assessed. RBP4 also showed selected renal associations, consistent with broader metabolic and organ stress. Leptin was reduced at admission but showed fewer and less consistent inflammatory and renal associations, and its trajectory may have been influenced by metabolic, nutritional, and treatment-related factors [16, 18].

CT-derived WHR demonstrated limited associations with post-traumatic adipokine responses, and most WHR-stratified comparisons showed substantial overlap. Nominal WHR × sex interactions for adiponectin were observed at D1 and D5, consistent with established sex-related differences in body-fat distribution and adiponectin biology [39, 40]; however, only 15 women were included in this study, and the interaction estimates were rather imprecise. These findings are therefore hypothesis-generating and do not establish a reproducible effect of WHR or sex on post-traumatic adipokine responses.

From a translational perspective, resistin and R/A are unlikely to replace established trauma measures but may warrant evaluation as complementary markers of the post-traumatic inflammatory–renal axis. ISS reflects anatomical injury burden, shock ratio and lactate reflects early circulatory and metabolic disturbance, SOFA quantifies established organ dysfunction [4], and IL-6 reflects systemic inflammation after major trauma [41]. Whether resistin or R/A adds clinically useful information beyond these measures remains undetermined at this point because no integrated multivariable prediction model was developed or validated.

Resistin and adiponectin are measurable using commercially available ELISA-based assays and, in standard laboratory settings, can be quantified within several hours, although measurements in the present study were research-based and not available in real time. Clinical translation would require prospective multicentre validation, demonstration of incremental value in internally and externally validated multivariable models, and standardized assays with reproducible turnaround times and clinically actionable cutoffs; until then, resistin and R/A should be considered research biomarkers.

Several limitations should be considered. This was a single-centre, predominantly male, middle-aged trauma cohort that excluded patients expected to survive < 24 h, limiting generalisability to the full spectrum of severe trauma. Injury patterns were heterogeneous; although AIS-region sensitivity analyses did not identify a single anatomical region accounting for the admission adipokine associations after FDR correction (Supplementary Table 4A), more specific injury phenotypes could not be resolved. Later timepoints were increasingly affected by death, discharge, and unavailable samples; therefore, D5 and D10 findings reflect observed contributors rather than the complete admission cohort. Subgroup, interaction, and ROC analyses, particularly the WHR × sex analyses and late mortality estimates, were exploratory and underpowered.

Metabolic, nutritional, treatment-related, and dilutional influences could not be fully separated from trauma-associated biology [16, 18, 42]. Fasting, nutritional status, and relevant treatment exposures were not systematically available, and early concentrations may have been affected by resuscitation-related hemodilution. Although hematocrit adjustment did not materially alter the strongest admission correlations (Supplementary Fig. 2B), it cannot distinguish biological from dilutional effects on absolute concentrations. Adipokine trajectories should therefore be interpreted as clinical-course-associated circulating patterns rather than direct measures of adipose-tissue secretion. Finally, CT-derived WHR is an indirect measure of central body-fat distribution rather than a direct measure of visceral adiposity [21].

## Conclusion

Severe trauma was associated with rapid changes in circulating adipokine profiles, characterised by early resistin elevation, adiponectin suppression, and an increased R/A ratio. Resistin and the R/A ratio showed the most consistent associations with systemic inflammation and organ dysfunction, particularly renal markers and selected SOFA domains. WHR-stratified differences were limited. Because this was an observational correlational analysis, these findings do not establish causality, temporal precedence, or validated prognostic utility. Larger prospective multicenter studies are required to validate these associations and determine whether adipokine-based indices provide clinically useful information beyond established clinical parameters.

## Supplementary Information

Below is the link to the electronic supplementary material.

Supplementary Material 1

## Data Availability

All data generated or analysed during this study are included in this published article and its supplementary information files. Further information can be provided by the corresponding author on reasonable request.

## Abbreviations

L/A ratio: Leptin-to-Adiponectin ratio
R/A ratio: Resistin-to-Adiponectin ratio
AUC: Area under the curve
BE: Base excess
CRP: C-reactive protein
CT-scan: Body computed tomography scan
D: Day
DAMP: Damage-associated molecular pattern
GCS: Glasgow Coma Scale
GFR: Glomerular filtration rate
h: Hours
HS: Hemorrhagic shock
HV: Healthy volunteers
ICU: Intensive Care Unit
IL-6: Interleukin-6
ISS: Injury Severity Score
LOS: Length of the hospital stay
MODS: Multi Organ Dysfunction Syndrome
pRBC: Packed red blood cell concentrates
PT: Polytrauma
RBP4: Retinol-binding protein 4
ROC: Receiver-operating characteristics
SOFA score: Sequential Organ Failure Assessment score
T2DM: Type 2 diabetes mellitus
WAT: White adipose tissue
WHR: Waist-to-hip ratio
