# Supplementary material for: Inflammation-linked adipokine profiles associate with acute organ dysfunction after severe trauma
Source: J Transl Med. 2026 Sep 10;24:1176. doi: 10.1186/s12967-026-08968-4 (PMC13570505; doi:10.1186/s12967-026-08968-4)
Supplement: Supplementary file 1 [file 12967_2026_8968_MOESM1_ESM.docx]

**SUPPLEMENTARY Materials**

**Extended inclusion and exclusion criteria**

All patients aged 18 years or older with an ISS ≥16 were eligible for inclusion. Patients were required to have undergone whole-body CT imaging on admission, have at least one plasma sample available within the D0–D1 interval, and have sufficient clinical data to enable reconstruction of SOFA scores. Complete D0–D10 sampling was not required for inclusion. Exclusion criteria included pregnancy, confirmed HIV infection, cardiogenic shock as primary diagnosis, known hematologic malignancy, cytotoxic chemotherapy in the preceding six months, and any rapidly progressive or terminal illness associated with an expected survival of less than 24 hours. Patients lacking adequate CT imaging for WHR measurement or with insufficient serial laboratory data for analysis were also excluded.

**Blood sampling and processing**

Blood samples were collected at four predefined time points after admission: within 4 hours of injury (D0), at 24 hours (D1), day 5 (D5), and day 10 (D10), each with a permitted deviation of ±10%. At D0, samples were primarily drawn from central venous catheters placed during initial resuscitation in the emergency department. Subsequent samples (D1–D10) were obtained either from arterial lines during ICU treatment or from peripheral venipuncture on the general ward. Blood was collected into EDTA tubes (Sarstedt, Nümbrecht, Germany) and processed according to standardized biobanking procedures. Within 30 minutes of collection, samples were centrifuged at 2,200 × g for 15 minutes at 4 °C. Plasma was aliquoted into polypropylene tubes and stored at -80 °C until analysis. All samples used for adipokine measurement were thawed only once, and no freeze-thaw cycles occurred before testing.

**Clinical laboratory measurements**

All routine clinical chemistry and hematology analyses were performed under accredited quality control procedures at the Central Laboratory of Clinical Chemistry, University Hospital Ulm. Interleukin-6 (IL-6) was quantified by electrochemiluminescence immunoassay using the Cobas 8000 e801 analyzer (Roche Diagnostics, Mannheim, Germany). C-reactive protein (CRP) and bilirubin levels were determined by turbidimetric and photometric assays, respectively, on the Cobas c system. Blood gas parameters including glucose, lactate, and base excess were obtained using Radiometer ABL825 or ABL800 FLEX analyzers (Radiometer, Copenhagen, Denmark). Platelet counts were measured as part of the complete blood count using the Sysmex XN-series hematology analyzer (Sysmex, Kobe, Japan). Creatinine was assessed using an enzymatic assay traceable to IDMS calibration standards**.**

**Adipokine ELISA procedures**

Plasma concentrations of retinol-binding protein-4 (RBP4), adiponectin, leptin, and resistin were measured using commercially available DuoSet ELISA kits (R&D Systems, Bio-Techne, Minneapolis, MN, USA) following the manufacturer’s instructions. The analytical ranges were 23.4–1,500 pg/mL for RBP4, 62.5–4,000 pg/mL for adiponectin, 31.2–2,000 pg/mL for leptin, and 31.2–2,000 pg/mL for resistin. All samples and standards were measured duplicate, and plates were read at 450 nm with wavelength correction at 540 nm. Intra-assay and inter-assay coefficients of variation remained below 10% for all assays. Sample dilutions were performed to ensure that all readings fell within the linear portion of the standard curve. The same assay platform was used for all samples to minimize inter-platform variability within the cohort.

**Reconstruction of SOFA scores and organ-specific subscores**

SOFA scores were reconstructed retrospectively for each sampling time point using all available clinical and laboratory data. For the neurological score, Glasgow Coma Scale (GCS) values at admission were extracted directly from emergency department records. At later time points, neurological assessments were obtained from ICU documentation; when Richmond Agitation-Sedation Scale (RASS) values were available, these were converted to GCS equivalents according to the published conversion method (24).

Hepatic SOFA scores were based on serum bilirubin. In cases where bilirubin results were missing at a given time point, the last available bilirubin measurement was carried forward to the next time-point. Respiratory SOFA scores were calculated using PaO₂/FiO₂ ratios derived from arterial blood gas analyses, with FiO₂ estimated from ventilator settings or high-flow oxygen devices. Cardiovascular SOFA scores were determined from vasoactive medication requirements and mean arterial pressure values. Renal SOFA scores incorporated both serum creatinine and, when available, urine output; creatinine values closest to the sampling time point were used consistently. Coagulation SOFA scores were based on platelet counts. All steps of the SOFA reconstruction process followed established scoring definitions and internal standard operating procedures.

**CT-based assessment of waist-to-hip ratio**

Waist-to-hip ratio (WHR) was measured from admission CT scans performed as part of the initial trauma evaluation. Waist circumference was measured on axial slices at the level of the umbilicus, while hip circumference was measured at the level of the femoral neck. All measurements were performed using ZEN 3.0 software (Carl Zeiss Microscopy). The CT-based approach enabled standardized measurement of waist and hip circumferences from admission imaging. WHR was used as an indirect proxy of body-fat distribution and was not considered a direct measure of visceral adipose tissue.

**Supplementary Table 1.** Baseline characteristics according to availability of D10 adipokine measurements

| **Baseline variable** | **D10 adipokine measurement available (n=48)** | **No D10 adipokine measurement (n=19)** |
| --- | --- | --- |
| Age, years | 47.5 [32.8–58.0] | 51.0 [39.0–65.5] |
| Female sex, n (%) | 11 (22.9%) | 4 (21.1%) |
| ISS | 27 [22–31] | 25.5 [17–36.5] (n=18) |
| Shock ratio | 1.22 [0.90–1.42] (n=47) | 0.95 [0.79–1.08] |
| Admission SOFA | 6 [4–8] | 6 [4–8] |
| Creatinine, µmol/L | 93 [73–117.2] | 85 [68.5–98.5] |
| GFR, mL/min | 62 [48.5–93] (n=35) | 82 [56.8–103.2] (n=16) |
| Resistin D0, ng/mL | 22.15 [13.90–30.36] (n=46) | 22.32 [11.27–35.41] |
| Adiponectin D0, µg/mL | 2.09 [1.38–3.97] (n=47) | 2.28 [1.77–3.31] |
| R/A D0 | 9.72 [4.20–17.87] (n=45) | 8.49 [4.58–14.83] |

**Values are median [IQR] unless otherwise stated. Patients were classified according to whether at least one adipokine measurement was available at D10. Sample size varies because of variable-specific missing data. The comparison was descriptive; no inferential hypothesis testing was performed because its purpose was to characterize potential attrition-related selection rather than to test equivalence between groups.*

**Supplementary Table 2.** Full correlation matrix adipokines × organ dysfunction scores. Values represent partial correlation coefficients (r) adjusted as in the main analysis. Partial correlations were adjusted for age, sex, and BMI.

| **Adipokine / Ratio** | **Scores** | **Shock ratio** | **ISS** | **SOFA-Score** | | | | **Cardiovascular Score** | | | | **Coagulation Score** | | | | **Liver Score** | | | | **Renal Creatinin Score** | | | | **Respiration Score** | | | |
| --- | --- | --- | --- | --- | --- | --- | --- | --- | --- | --- | --- | --- | --- | --- | --- | --- | --- | --- | --- | --- | --- | --- | --- | --- | --- | --- | --- |
|  | **Day** | **D0** | **D0** | **D0** | **D1** | **D5** | **D10** | **D0** | **D1** | **D5** | **D10** | **D0** | **D1** | **D5** | **D10** | **D0** | **D1** | **D5** | **D10** | **D0** | **D1** | **D5** | **D10** | **D0** | **D1** | **D5** | **D10** |
| **Adiponectin (ug/ml)** | **D0** | -0.27* | 0.05 | -0.22 | -0.22 | -0.15 | -0.34* | -0.16 | -0.15 | -0.22 | -0.24 | 0.01 | 0.05 | 0.14 | 0.18 | -0.06 | -0.12 | -0.09 | -0.15 | -0.32* | -0.35** | -0.25 | -0.33* | -0.28* | -0.14 | -0.18 | -0.27 |
|  | **D1** | -0.04 | 0.27* | 0.11 | -0.01 | -0.15 | -0.26 | 0.18 | 0.09 | -0.13 | -0.12 | 0.28* | 0.17 | 0.12 | 0.19 | -0.15 | 0.05 | -0.04 | -0.06 | -0.12 | -0.15 | 0 | -0.30* | -0.2 | -0.16 | -0.14 | -0.23 |
|  | **D5** | -0.23 | 0.02 | -0.14 | -0.29* | -0.44** | -0.50*** | -0.13 | -0.04 | -0.28* | -0.33* | 0.26 | 0.09 | -0.07 | 0.02 | -0.02 | 0.1 | -0.23 | -0.09 | -0.29* | -0.35** | -0.26 | -0.40** | -0.33* | -0.34* | -0.28* | -0.31* |
|  | **D10** | -0.13 | -0.05 | -0.27 | -0.17 | -0.24 | -0.40** | -0.08 | 0.05 | -0.1 | -0.21 | 0.18 | 0.21 | 0.15 | -0.21 | -0.2 | -0.06 | -0.37* | -0.26 | -0.1 | -0.18 | -0.09 | -0.38* | -0.40** | -0.30* | -0.19 | -0.29* |
| **Leptin**  **(ng/ml)** | **D0** | 0.29* | -0.14 | -0.07 | -0.06 | -0.08 | 0.05 | -0.02 | -0.15 | -0.2 | 0.01 | -0.08 | -0.18 | 0 | -0.06 | -0.09 | -0.02 | 0.1 | 0.07 | 0.11 | 0.28* | 0.15 | 0.39** | 0.32* | 0.13 | 0.29* | 0.04 |
|  | **D1** | 0.22 | -0.07 | 0.03 | 0 | 0.03 | 0.14 | -0.01 | -0.11 | -0.12 | 0.01 | -0.12 | -0.15 | 0.06 | -0.15 | -0.15 | 0.06 | 0.02 | 0.03 | 0.17 | 0.27* | 0.41** | 0.40** | 0.21 | 0.11 | 0.24 | 0.21 |
|  | **D5** | 0.16 | 0.16 | 0.18 | 0.21 | 0.23 | 0.37* | -0.01 | 0.11 | 0.12 | 0.21 | -0.18 | -0.19 | 0.09 | 0.08 | -0.09 | -0.02 | 0.13 | 0.04 | 0.15 | 0.31* | 0.33* | 0.45** | 0.25 | 0.27 | 0.22 | 0.36* |
|  | **D10** | 0.19 | -0.05 | 0.12 | 0.19 | 0.09 | 0.33* | 0.04 | 0.01 | -0.02 | 0.23 | 0.08 | -0.17 | -0.01 | -0.06 | -0.35* | -0.03 | -0.11 | -0.24 | 0.23 | 0.47** | 0.31* | 0.41** | 0.2 | 0.05 | 0 | 0.15 |
| **Resistin (ng/ml)** | **D0** | 0.23 | -0.04 | 0.13 | 0.08 | 0.05 | 0.12 | 0.11 | -0.19 | -0.12 | 0.05 | -0.11 | 0.19 | 0.07 | -0.02 | 0.07 | 0.1 | 0.30* | 0.14 | 0.08 | 0.24 | 0.1 | 0.39** | 0.36** | 0.09 | 0.05 | 0.06 |
|  | **D1** | 0.62*** | 0.1 | 0.36** | 0.42** | 0.31* | 0.09 | 0.44*** | 0.24 | 0.15 | -0.02 | -0.07 | 0.17 | 0.03 | -0.23 | 0.05 | 0.35** | 0.05 | 0.17 | 0.29* | 0.47*** | 0.53*** | 0.27 | 0.19 | 0.18 | 0.31* | 0.05 |
|  | **D5** | 0.24 | -0.1 | 0.1 | 0.11 | 0.06 | 0.14 | 0.17 | 0.01 | 0.13 | 0.14 | 0.01 | 0.03 | -0.07 | 0.04 | 0.16 | 0.24 | 0.32* | 0.23 | 0.04 | 0.18 | 0.08 | 0.18 | 0.2 | 0.18 | 0.19 | 0.23 |
|  | **D10** | 0.24 | -0.18 | 0 | 0.38* | 0.45** | 0.57*** | 0.03 | 0.22 | 0.59*** | 0.51*** | 0.11 | 0.09 | -0.1 | -0.16 | 0.03 | -0.02 | 0.15 | 0.05 | 0.13 | 0.2 | 0.34* | 0.32* | 0.2 | 0.31* | 0.40** | 0.58*** |
| **RBP4**  **(ug/ml)** | **D0** | -0.06 | -0.21 | -0.33* | -0.43*** | -0.22 | -0.17 | -0.44*** | -0.35** | -0.23 | -0.04 | -0.13 | -0.04 | 0.05 | 0.11 | 0 | -0.34** | -0.05 | 0.01 | 0.05 | -0.09 | -0.12 | 0.11 | 0.15 | -0.34** | -0.05 | -0.17 |
|  | **D1** | 0.21 | -0.15 | -0.03 | -0.06 | -0.05 | 0.04 | -0.05 | -0.24 | -0.2 | 0.14 | -0.26 | 0.11 | 0.13 | -0.08 | -0.12 | -0.27* | -0.02 | 0.02 | 0.53*** | 0.48*** | 0.30* | 0.31* | 0.31* | -0.15 | -0.13 | -0.16 |
|  | **D5** | 0.16 | 0.1 | 0.15 | 0.06 | -0.06 | 0.01 | -0.08 | -0.09 | -0.31* | -0.06 | -0.17 | -0.13 | 0 | -0.13 | 0.03 | 0.08 | -0.05 | -0.01 | 0.21 | 0.26 | 0.19 | 0.28 | 0.19 | 0 | -0.23 | -0.08 |
|  | **D10** | 0.34* | -0.19 | 0.12 | 0.13 | 0.19 | 0.03 | 0.19 | -0.02 | 0.03 | 0.06 | -0.32* | -0.09 | -0.02 | -0.41** | -0.09 | -0.18 | -0.06 | -0.02 | 0.45** | 0.35* | 0.55*** | 0.42** | 0.31* | 0.01 | 0.02 | -0.18 |
| **Leptin/Adiponectin ratio** | **D0** | 0.30* | -0.19 | -0.02 | -0.01 | -0.05 | 0.19 | 0.01 | -0.09 | -0.12 | 0.1 | -0.06 | -0.2 | -0.1 | -0.13 | -0.09 | 0.04 | 0.07 | 0.05 | 0.19 | 0.38** | 0.13 | 0.40** | 0.30* | 0.11 | 0.33* | 0.17 |
|  | **D1** | 0.28 | -0.19 | -0.01 | 0 | 0.07 | 0.24 | -0.05 | -0.13 | -0.07 | 0.13 | -0.26 | -0.16 | -0.02 | -0.25 | -0.1 | -0.01 | -0.03 | 0.02 | 0.27 | 0.31* | 0.37** | 0.47*** | 0.28* | 0.15 | 0.29* | 0.28 |
|  | **D5** | 0.34* | 0.16 | 0.22 | 0.26 | 0.34* | 0.42** | 0.15 | 0.08 | 0.18 | 0.27 | -0.30* | -0.21 | 0.09 | 0.07 | -0.1 | -0.06 | 0.18 | 0.03 | 0.31* | 0.40** | 0.37** | 0.50*** | 0.34* | 0.38** | 0.29* | 0.36* |
|  | **D10** | 0.3 | 0 | 0.23 | 0.17 | 0.17 | 0.45** | 0.16 | -0.04 | 0.05 | 0.30* | -0.11 | -0.29 | -0.13 | 0.05 | -0.19 | 0 | 0.09 | -0.08 | 0.23 | 0.42** | 0.30* | 0.50*** | 0.37* | 0.15 | 0.11 | 0.24 |
| **Resistin/Adiponectin ratio** | **D0** | 0.32* | -0.06 | 0.18 | 0.16 | 0.1 | 0.26 | 0.19 | -0.1 | -0.05 | 0.17 | -0.17 | 0.08 | -0.01 | -0.1 | 0.05 | 0.13 | 0.25 | 0.15 | 0.23 | 0.39** | 0.14 | 0.45** | 0.44*** | 0.17 | 0.13 | 0.15 |
|  | **D1** | 0.55*** | -0.11 | 0.12 | 0.21 | 0.25 | 0.16 | 0.31* | 0.1 | 0.17 | 0.1 | -0.33* | 0.05 | -0.09 | -0.29 | 0.09 | 0.12 | 0.1 | 0.17 | 0.31* | 0.39** | 0.34* | 0.26 | 0.26 | 0.15 | 0.32* | 0.16 |
|  | **D5** | 0.2 | -0.1 | 0.07 | 0.19 | 0.24 | 0.29* | 0.12 | -0.01 | 0.13 | 0.13 | -0.14 | -0.1 | 0.04 | -0.04 | 0.12 | 0.08 | 0.31* | 0.19 | 0.13 | 0.36* | 0.07 | 0.37* | 0.25 | 0.26 | 0.23 | 0.26 |
|  | **D10** | 0.26 | -0.13 | 0.13 | 0.34* | 0.45** | 0.60*** | 0.12 | 0.11 | 0.41** | 0.46** | -0.23 | -0.15 | -0.26 | -0.07 | 0.15 | -0.03 | 0.35* | 0.21 | 0.17 | 0.21 | 0.24 | 0.47** | 0.38* | 0.37* | 0.42** | 0.50*** |

* Values represent partial Spearman correlation coefficients adjusted for age, sex, and BMI. Benjamini–Hochberg correction was applied across the predefined correlation family, and asterisks indicate FDR-adjusted significance: *q<0.05, **q<0.01, ***q<0.001. All coefficients are shown for completeness; only associations with q<0.05 and |r|≥0.30 were interpreted in the manuscript.

**Supplementary Table 3.** Full correlation matrix adipokines × inflammation and kidney function markers. Values represent partial correlation coefficients (r) adjusted as in the main analysis. Partial correlations were adjusted for age, sex, and BMI.

|  | **Markers** | **Glucose (mg/dl)** | **Lactate** | **BE** | **IL-6 (pg/ml)** | **CRP (mg/l)** | | | | **GFR (ml/min)** | | | | **Creatinin (µmol/l)** | | | |
| --- | --- | --- | --- | --- | --- | --- | --- | --- | --- | --- | --- | --- | --- | --- | --- | --- | --- |
|  | **Days** | **D0** | **D0** | **D0** | **D0** | **D0** | **D1** | **D5** | **D10** | **D0** | **D1** | **D5** | **D10** | **D0** | **D1** | **D5** | **D10** |
| **Adiponectin (ug/ml)** | D0 | 0.08 | -0.18 | 0.26* | -0.3 | 0.14 | -0.19 | -0.24 | -0.36** | 0.11 | 0.16 | 0.06 | 0.32* | -0.27* | -0.27* | -0.27* | -0.24 |
|  | D1 | 0.13 | -0.01 | 0.03 | -0.16 | -0.08 | -0.07 | -0.28* | -0.32* | 0.01 | 0.03 | -0.03 | 0.13 | -0.15 | -0.16 | -0.29* | -0.42** |
|  | D5 | -0.22 | -0.16 | 0.30* | -0.29 | -0.03 | -0.12 | -0.37** | -0.40** | 0.21 | 0.35* | 0.37* | 0.33* | -0.33* | -0.47*** | -0.39** | -0.52*** |
|  | D10 | -0.11 | 0.05 | 0.24 | 0.02 | -0.05 | 0.12 | -0.39** | -0.35* | 0.17 | 0.03 | 0.05 | 0.14 | -0.16 | -0.33* | -0.32* | -0.44** |
| **Leptin (ng/ml)** | D0 | 0.07 | -0.13 | 0.03 | 0.32 | 0.38** | -0.04 | 0.04 | -0.02 | -0.08 | -0.08 | -0.09 | -0.29 | 0.04 | -0.03 | 0.2 | 0.08 |
|  | D1 | 0.08 | -0.18 | -0.11 | -0.23 | 0.2 | -0.01 | 0.05 | 0.05 | -0.09 | -0.07 | -0.03 | -0.48** | 0.05 | 0 | 0.34* | 0.23 |
|  | D5 | 0.28* | 0.04 | -0.19 | 0.05 | 0.29* | 0.12 | 0.28* | 0.13 | -0.14 | -0.24 | -0.24 | -0.46** | 0.06 | 0.09 | 0.39** | 0.23 |
|  | D10 | 0.07 | 0.07 | -0.07 | -0.03 | 0.06 | -0.03 | -0.01 | 0.12 | -0.01 | -0.3 | -0.26 | -0.50** | 0.09 | 0.17 | 0.29 | 0.07 |
| **Resistin (ng/ml)** | D0 | 0.21 | 0.24 | 0.09 | 0.75*** | 0.30* | -0.03 | 0.03 | 0.04 | -0.03 | -0.14 | -0.2 | -0.12 | 0.13 | 0.26* | 0.2 | 0.27 |
|  | D1 | 0.31* | 0.38* | -0.32* | 0.54** | 0.07 | 0.28* | 0.27 | 0.09 | -0.38* | -0.52** | -0.50** | -0.55*** | 0.39** | 0.42** | 0.46*** | 0.27 |
|  | D5 | 0 | 0.40* | -0.11 | 0.65** | 0.04 | 0.15 | 0.24 | 0.29 | -0.14 | 0.08 | -0.04 | -0.02 | 0.1 | 0.19 | 0.24 | 0.1 |
|  | D10 | 0.1 | 0.21 | -0.01 | 0.34 | 0.03 | 0.24 | 0.22 | 0.3 | -0.07 | 0.05 | -0.05 | -0.47* | 0.13 | 0.22 | 0.40** | 0.43** |
| **RBP4 (ug/ml)** | D0 | 0.05 | -0.22 | 0.27* | -0.34 | 0.04 | -0.26* | -0.06 | -0.26 | -0.04 | 0.13 | 0.07 | -0.02 | -0.01 | -0.13 | 0.03 | 0.13 |
|  | D1 | 0.26 | 0.09 | -0.04 | 0.03 | -0.09 | -0.18 | -0.16 | -0.17 | -0.26 | -0.47** | -0.47** | -0.36* | 0.42** | 0.41** | 0.33* | 0.44** |
|  | D5 | -0.01 | 0.27 | -0.09 | 0.08 | -0.12 | -0.21 | -0.23 | -0.29* | -0.05 | -0.08 | 0.07 | -0.04 | 0.24 | 0.16 | 0.37** | 0.38** |
|  | D10 | 0.21 | 0.08 | -0.21 | 0.44* | 0.01 | 0.1 | -0.05 | -0.34* | -0.23 | -0.48** | -0.43* | -0.58*** | 0.43** | 0.27 | 0.63*** | 0.44** |
| **Leptin/Adiponectin ratio** | D0 | -0.05 | -0.09 | -0.05 | 0.22 | 0.28* | 0.08 | 0.11 | 0.17 | -0.07 | -0.09 | -0.05 | -0.34* | 0.09 | 0.09 | 0.2 | 0.12 |
|  | D1 | 0.07 | -0.17 | -0.12 | -0.18 | 0.23 | 0.01 | 0.17 | 0.11 | -0.13 | -0.12 | -0.07 | -0.53*** | 0.16 | 0.1 | 0.42** | 0.39** |
|  | D5 | 0.38** | 0.13 | -0.39** | 0.03 | 0.19 | 0.21 | 0.48*** | 0.29 | -0.28 | -0.44** | -0.45** | -0.53*** | 0.28* | 0.36* | 0.50*** | 0.40** |
|  | D10 | 0.11 | 0.07 | -0.24 | -0.01 | 0.05 | -0.03 | 0.25 | 0.22 | -0.13 | -0.25 | -0.26 | -0.47** | 0.18 | 0.29 | 0.39** | 0.28 |
| **Resistin/Adiponectin ratio** | D0 | 0.16 | 0.27 | -0.12 | 0.65*** | 0.19 | 0.06 | 0.13 | 0.28 | -0.15 | -0.37* | -0.27 | -0.33* | 0.27* | 0.36** | 0.29* | 0.37** |
|  | D1 | 0.34* | 0.19 | -0.27 | 0.64*** | 0.17 | 0.21 | 0.32* | 0.18 | -0.31 | -0.45** | -0.36* | -0.51** | 0.41** | 0.39** | 0.46*** | 0.41** |
|  | D5 | 0.06 | 0.3 | -0.25 | 0.59** | 0.09 | 0.09 | 0.37** | 0.46** | -0.26 | -0.33 | -0.38* | -0.41* | 0.23 | 0.40** | 0.36* | 0.35* |
|  | D10 | 0.17 | 0.07 | -0.19 | 0.2 | 0.1 | 0.03 | 0.42** | 0.36* | -0.33 | -0.08 | -0.14 | -0.43* | 0.24 | 0.32* | 0.49*** | 0.55*** |

*** *Values represent partial Spearman correlation coefficients adjusted for age, sex, and BMI. Benjamini–Hochberg correction was applied across the predefined correlation family, and asterisks indicate FDR-adjusted significance: *q<0.05, **q<0.01, ***q<0.001. All coefficients are shown for completeness; only associations with q<0.05 and |r|≥0.30 were interpreted in the manuscript.*

**Supplementary Table 4A. Admission AIS injury-site sensitivity analysis.** Partial Spearman correlations between admission (D0) circulating markers and AIS body-region scores, adjusted for age, sex, BMI, and global ISS.

| **D0 marker** | **AIS region** | **n** | **partial r** | **p-value** | **FDR q** |
| --- | --- | --- | --- | --- | --- |
| **Adiponectin** | Head | 60 | 0.22 | 0.096 | 0.408 |
|  | Face | 60 | -0.29 | 0.029 | 0.207 |
|  | Chest | 60 | 0.01 | 0.935 | 0.990 |
|  | Abdomen | 60 | -0.14 | 0.321 | 0.729 |
|  | Extremities/Pelvic | 60 | -0.16 | 0.240 | 0.664 |
|  | External | 60 | -0.11 | 0.401 | 0.729 |
| **Leptin** | Head | 55 | -0.36 | 0.009 | 0.139 |
|  | Face | 55 | -0.10 | 0.480 | 0.729 |
|  | Chest | 55 | 0.25 | 0.080 | 0.408 |
|  | Abdomen | 55 | -0.01 | 0.963 | 0.990 |
|  | Extremities/Pelvic | 55 | 0.20 | 0.151 | 0.532 |
|  | External | 55 | 0.04 | 0.796 | 0.924 |
| **Resistin** | Head | 59 | -0.19 | 0.163 | 0.532 |
|  | Face | 59 | -0.09 | 0.503 | 0.729 |
|  | Chest | 59 | 0.07 | 0.592 | 0.790 |
|  | Abdomen | 59 | 0.05 | 0.733 | 0.909 |
|  | Extremities/Pelvic | 59 | 0.24 | 0.083 | 0.408 |
|  | External | 59 | -0.06 | 0.681 | 0.876 |
| **RBP4** | Head | 59 | 0.00 | 0.993 | 0.993 |
|  | Face | 59 | -0.16 | 0.236 | 0.664 |
|  | Chest | 59 | 0.11 | 0.438 | 0.729 |
|  | Abdomen | 59 | -0.12 | 0.386 | 0.729 |
|  | Extremities/Pelvic | 59 | -0.13 | 0.357 | 0.729 |
|  | External | 59 | -0.02 | 0.895 | 0.990 |
| **L/A ratio** | Head | 55 | -0.39 | 0.004 | 0.139 |
|  | Face | 55 | 0.01 | 0.918 | 0.990 |
|  | Chest | 55 | 0.15 | 0.295 | 0.729 |
|  | Abdomen | 55 | 0.09 | 0.510 | 0.729 |
|  | Extremities/Pelvic | 55 | 0.23 | 0.102 | 0.408 |
|  | External | 55 | 0.11 | 0.445 | 0.729 |
| **R/A ratio** | Head | 59 | -0.31 | 0.022 | 0.200 |
|  | Face | 59 | 0.10 | 0.480 | 0.729 |
|  | Chest | 59 | 0.04 | 0.771 | 0.924 |
|  | Abdomen | 59 | 0.12 | 0.380 | 0.729 |
|  | Extremities/Pelvic | 59 | 0.34 | 0.012 | 0.139 |
|  | External | 59 | 0.09 | 0.527 | 0.729 |

**Values represent partial Spearman correlation coefficients adjusted for age, sex, BMI, and global ISS. Benjamini–Hochberg FDR correction was applied across all 36 AIS region–marker comparisons. No association remained significant after FDR correction (all q>0.05). Sample size varies according to availability of the corresponding D0 biomarker measurement.*

**Supplementary Table 4B. Baseline-adjusted lagged associations between admission adipokines and subsequent renal dysfunction.** Partial Spearman correlations of D0 resistin, adiponectin, and R/A with renal SOFA, creatinine, and GFR at D1 and D5, adjusted for age, sex, BMI, and the corresponding D0 renal measure. Benjamini–Hochberg FDR correction was applied across all 18 comparisons.

| **D0 marker** | **Later renal outcome** | **Baseline adjustment** | **n** | **partial r** | **p** | **FDR q** |
| --- | --- | --- | --- | --- | --- | --- |
| Resistin D0 | Renal SOFA D1 | Renal SOFA D0 | 58 | 0.23 | 0.092 | 0.268 |
|  | Renal SOFA D5 | Renal SOFA D0 | 53 | 0.09 | 0.524 | 0.674 |
|  | Creatinine D1 | Creatinine D0 | 59 | 0.22 | 0.111 | 0.268 |
|  | Creatinine D5 | Creatinine D0 | 55 | 0.15 | 0.303 | 0.455 |
|  | GFR D1 | GFR D0 | 38 | -0.21 | 0.245 | 0.438 |
|  | GFR D5 | GFR D0 | 39 | -0.30 | 0.078 | 0.268 |
| Adiponectin D0 | Renal SOFA D1 | Renal SOFA D0 | 59 | -0.23 | 0.092 | 0.268 |
|  | Renal SOFA D5 | Renal SOFA D0 | 54 | -0.05 | 0.735 | 0.826 |
|  | Creatinine D1 | Creatinine D0 | 59 | -0.17 | 0.225 | 0.438 |
|  | Creatinine D5 | Creatinine D0 | 56 | -0.13 | 0.356 | 0.492 |
|  | GFR D1 | GFR D0 | 39 | 0.06 | 0.713 | 0.826 |
|  | GFR D5 | GFR D0 | 40 | -0.01 | 0.937 | 0.937 |
| R/A D0 | Renal SOFA D1 | Renal SOFA D0 | 58 | 0.33 | 0.014 | 0.258 |
|  | Renal SOFA D5 | Renal SOFA D0 | 53 | 0.03 | 0.850 | 0.900 |
|  | Creatinine D1 | Creatinine D0 | 59 | 0.25 | 0.066 | 0.268 |
|  | Creatinine D5 | Creatinine D0 | 55 | 0.16 | 0.268 | 0.438 |
|  | GFR D1 | GFR D0 | 38 | -0.34 | 0.053 | 0.268 |
|  | GFR D5 | GFR D0 | 39 | -0.27 | 0.119 | 0.268 |

**Values are partial Spearman correlation coefficients (r) based on available complete observations; n therefore varies across analyses. No association remained significant after FDR correction (all q>0.05).*

# **Supplementary Table 5A. ROC performance of adipokines.** Exploratory ROC analysis of raw adipokine concentrations (D0, D1, D5, D10) for hospital length of stay (LOS), ICU LOS, shock severity (shock ratio), and 30-day mortality.

| **Outcome** | **Marker** | **Day** | **AUC** | **95% CI** | **p-value** | **Sig** |
| --- | --- | --- | --- | --- | --- | --- |
| LOS Hospital | Adiponectin | D0 | 0.32 | 0.18–0.48 | 0.025 | * |
|  | Adiponectin | D1 | 0.36 | 0.22–0.51 | 0.087 |  |
|  | Adiponectin | D5 | 0.34 | 0.20–0.49 | 0.049 | * |
|  | Adiponectin | D10 | 0.25 | 0.11–0.41 | 0.005 | * |
|  | Leptin | D0 | 0.51 | 0.32–0.67 | 0.960 |  |
|  | Leptin | D1 | 0.52 | 0.37–0.67 | 0.861 |  |
|  | Leptin | D5 | 0.63 | 0.45–0.78 | 0.116 |  |
|  | Leptin | D10 | 0.57 | 0.38–0.73 | 0.471 |  |
|  | Resistin | D0 | 0.50 | 0.33–0.66 | 1.000 |  |
|  | Resistin | D1 | 0.65 | 0.48–0.81 | 0.080 |  |
|  | Resistin | D5 | 0.56 | 0.40–0.73 | 0.475 |  |
|  | Resistin | D10 | 0.58 | 0.39–0.76 | 0.408 |  |
|  | RBP4 | D0 | 0.39 | 0.25–0.58 | 0.189 |  |
|  | RBP4 | D1 | 0.43 | 0.27–0.59 | 0.418 |  |
|  | RBP4 | D5 | 0.42 | 0.28–0.59 | 0.362 |  |
|  | RBP4 | D10 | 0.51 | 0.34–0.68 | 0.893 |  |
| LOS ICU | Adiponectin | D0 | 0.43 | 0.26–0.58 | 0.404 |  |
|  | Adiponectin | D1 | 0.43 | 0.28–0.59 | 0.419 |  |
|  | Adiponectin | D5 | 0.39 | 0.24–0.55 | 0.198 |  |
|  | Adiponectin | D10 | 0.42 | 0.25–0.61 | 0.392 |  |
|  | Leptin | D0 | 0.48 | 0.33–0.65 | 0.860 |  |
|  | Leptin | D1 | 0.49 | 0.29–0.68 | 0.910 |  |
|  | Leptin | D5 | 0.72 | 0.53–0.87 | 0.014 | * |
|  | Leptin | D10 | 0.59 | 0.41–0.77 | 0.348 |  |
|  | Resistin | D0 | 0.35 | 0.20–0.52 | 0.070 |  |
|  | Resistin | D1 | 0.54 | 0.40–0.70 | 0.625 |  |
|  | Resistin | D5 | 0.48 | 0.31–0.64 | 0.796 |  |
|  | Resistin | D10 | 0.64 | 0.47–0.81 | 0.147 |  |
|  | RBP4 | D0 | 0.51 | 0.35–0.68 | 0.902 |  |
|  | RBP4 | D1 | 0.53 | 0.34–0.71 | 0.751 |  |
|  | RBP4 | D5 | 0.46 | 0.29–0.64 | 0.621 |  |
|  | RBP4 | D10 | 0.50 | 0.31–0.68 | 0.969 |  |
| Shock ratio | Adiponectin | D0 | 0.35 | 0.22–0.49 | 0.039 | * |
|  | Leptin | D0 | 0.65 | 0.51–0.78 | 0.048 | * |
|  | Resistin | D0 | 0.58 | 0.45–0.71 | 0.286 |  |
|  | RBP4 | D0 | 0.51 | 0.36–0.67 | 0.904 |  |
| 30d mortality | Adiponectin | D0 | 0.52 | 0.36–0.70 | 0.815 |  |
|  | Adiponectin | D1 | 0.45 | 0.25–0.69 | 0.648 |  |
|  | Adiponectin | D5 | 0.30 | 0.07–0.56 | 0.086 |  |
|  | Adiponectin | D10 | 0.29 | 0.02–0.63 | 0.173 |  |
|  | Leptin | D0 | 0.36 | 0.18–0.55 | 0.152 |  |
|  | Leptin | D1 | 0.51 | 0.26–0.76 | 0.973 |  |
|  | Leptin | D5 | 0.54 | 0.32–0.76 | 0.748 |  |
|  | Leptin | D10 | 0.48 | 0.09–0.87 | 0.894 |  |
|  | Resistin | D0 | 0.64 | 0.45–0.81 | 0.125 |  |
|  | Resistin | D1 | 0.72 | 0.55–0.87 | 0.050 | * |
|  | Resistin | D5 | 0.77 | 0.60–0.92 | 0.077 |  |
|  | Resistin | D10 | 0.80 | 0.67–0.92 | 0.094 |  |
|  | RBP4 | D0 | 0.45 | 0.32–0.61 | 0.623 |  |
|  | RBP4 | D1 | 0.44 | 0.24–0.64 | 0.551 |  |
|  | RBP4 | D5 | 0.39 | 0.15–0.65 | 0.349 |  |
|  | RBP4 | D10 | 0.43 | 0.09–0.82 | 0.705 |  |

*For mortality (n=67 for D0, n=51 for D1 (with 7 death), n=47 for D5 (with 4 death), and n=42 for D10 (with 3 death). Values represent AUCs with 95% confidence intervals. P-values are from Mann-Whitney U tests comparing biomarker distributions between outcome groups and do not represent AUC comparisons. AUC values <0.5 indicate inverse discrimination.

**Supplementary Table 5B. ROC performance of adipokine ratios.** Exploratory ROC analysis of adipokine ratios (L/A and R/A) measured at D0, D1, D5, and D10 for hospital LOS, ICU LOS, shock severity, and 30-day mortality.

| **Outcome** | **Ratio** | **Day** | **AUC** | **95% CI** | **p-value** | **Sig** |
| --- | --- | --- | --- | --- | --- | --- |
| LOS Hospital | L/A | D0 | 0.59 | 0.41–0.74 | 0.284 |  |
|  | L/A | D1 | 0.59 | 0.46–0.75 | 0.260 |  |
|  | L/A | D5 | 0.70 | 0.54–0.85 | 0.016 | * |
|  | L/A | D10 | 0.67 | 0.50–0.83 | 0.063 |  |
|  | R/A | D0 | 0.62 | 0.44–0.78 | 0.135 |  |
|  | R/A | D1 | 0.67 | 0.51–0.82 | 0.041 | * |
|  | R/A | D5 | 0.62 | 0.45–0.79 | 0.156 |  |
|  | R/A | D10 | 0.71 | 0.53–0.86 | 0.028 | * |
| LOS ICU | L/A | D0 | 0.49 | 0.32–0.67 | 0.897 |  |
|  | L/A | D1 | 0.53 | 0.34–0.72 | 0.777 |  |
|  | L/A | D5 | 0.66 | 0.49–0.82 | 0.069 |  |
|  | L/A | D10 | 0.56 | 0.37–0.73 | 0.516 |  |
|  | R/A | D0 | 0.41 | 0.25–0.56 | 0.270 |  |
|  | R/A | D1 | 0.55 | 0.40–0.76 | 0.547 |  |
|  | R/A | D5 | 0.52 | 0.35–0.70 | 0.814 |  |
|  | R/A | D10 | 0.65 | 0.47–0.80 | 0.125 |  |
| Shock ratio | L/A | D0 | 0.69 | 0.57–0.81 | 0.012 | * |
|  | A/L | D0 | 0.31 | 0.18–0.43 | 0.011 | * |
|  | A/R | D0 | 0.34 | 0.22–0.47 | 0.028 | * |
|  | R/A | D0 | 0.66 | 0.53–0.79 | 0.030 | * |
| 30-day mortality | L/A | D0 | 0.35 | 0.17–0.55 | 0.121 |  |
|  | L/A | D1 | 0.50 | 0.21–0.80 | 1.000 |  |
|  | L/A | D5 | 0.60 | 0.37–0.83 | 0.408 |  |
|  | L/A | D10 | 0.55 | 0.24–0.98 | 0.749 |  |
|  | R/A | D0 | 0.58 | 0.42–0.74 | 0.398 |  |
|  | R/A | D1 | 0.71 | 0.52–0.89 | 0.070 |  |
|  | R/A | D5 | 0.88 | 0.72–1.00 | 0.013 | * |
|  | R/A | D10 | 0.86 | 0.63–1.00 | 0.043 | * |

* For mortality (n=67 for D0, n=51 for D1 (with 7 death), n=47 for D5 (with 4 death), and n=42 for D10 (with 3 death). Values represent AUCs with 95% confidence intervals. P-values are from Mann-Whitney U tests comparing biomarker distributions between outcome groups and do not represent AUC comparisons. AUC values <0.5 indicate inverse discrimination.

**Supplementary Table 5C. Exploratory ROC performance of established clinical variables.**

| **Outcome** | **Marker** | **n (events)** | **AUC** | **95% CI** | **p** |
| --- | --- | --- | --- | --- | --- |
| LOS >21 d | ISS | 54 (26) | 0.65 | 0.50–0.79 | 0.065 |
|  | Shock ratio | 53 (25) | 0.74 | 0.61–0.88 | 0.003 |
|  | Base excess | 53 (26) | 0.19 | 0.07–0.31 | <0.001 |
|  | Lactate | 40 (20) | 0.60 | 0.42–0.78 | 0.272 |
|  | IL-6 | 23 (16) | 0.71 | 0.50–0.93 | 0.118 |
|  | Admission SOFA | 54 (26) | 0.79 | 0.67–0.91 | <0.001 |
|  | Creatinine | 54 (26) | 0.67 | 0.53–0.81 | 0.032 |
|  | GFR | 41 (19) | 0.31 | 0.14–0.48 | 0.041 |
|  | CRP | 54 (26) | 0.49 | 0.33–0.65 | 0.937 |
| 30-day mortality | ISS | 66 (12) | 0.68 | 0.50–0.86 | 0.054 |
|  | Shock ratio | 66 (13) | 0.54 | 0.37–0.72 | 0.634 |
|  | Base excess | 65 (12) | 0.39 | 0.23–0.54 | 0.220 |
|  | Lactate | 50 (10) | 0.66 | 0.46–0.85 | 0.135 |
|  | IL-6 | 31 (8) | 0.48 | 0.21–0.74 | 0.877 |
|  | Admission SOFA | 67 (13) | 0.70 | 0.54–0.86 | 0.026 |
|  | Creatinine | 67 (13) | 0.50 | 0.31–0.70 | 0.981 |
|  | GFR | 51 (10) | 0.35 | 0.15–0.56 | 0.158 |
|  | CRP | 67 (13) | 0.59 | 0.39–0.79 | 0.326 |

*** *AUC 95% confidence intervals were estimated using DeLong's method. P-values are descriptive two-sided Mann–Whitney U tests, consistent with Supplementary Tables 5A and 5B. AUC <0.5 denotes inverse discrimination; no direction was reversed to maximize AUC. Analyses were performed using available observations for each variable, and n therefore varies across analyses.*

**Supplementary Figure**

**Supplementary Figure 1. Sex-specific WHR associations and interaction effects for adipokines.** (A) Scatterplots showing the association between WHR and adiponectin concentrations at D1 and D5, stratified by sex (male: blue; female: red). Linear regression lines are displayed separately for men and women. (B) Forest plot displaying interaction terms (β coefficients with 95% CIs) for the WHR × sex interaction across adipokines and adipokine ratios at D0, D1, D5 and D10. Positive values indicate greater WHR-associated change in females; negative values indicate greater change in males. The vertical dashed line represents no interaction effect. * p<0.05 indicates nominal statistical significance.

**Supplementary Figure 2. Incremental discrimination and admission hematocrit sensitivity.** (A) Paired differences in AUC (R/A minus resistin alone) for hospital LOS >21 days and 30-day mortality at D0, D1, D5, and D10, with 95% confidence intervals from the paired DeLong test. Paired comparisons are restricted to patients with both markers measured at the given timepoint and a confirmed ISS >= 16 (n=59, 51, 47 and 42 at D0, D1, D5 and D10). Positive values favor R/A. The 95% CI excluded zero only for the D0/hospital-LOS comparison (ΔAUC 0.13, 95% CI 0.01-0.26); the remaining seven of eight comparisons were consistent with no difference. (B) Partial Spearman correlation coefficients for 16 selected admission (D0) adipokine/ratio–clinical associations, adjusted for age, sex, and BMI before and after additional adjustment for admission hematocrit. Connecting lines show the change after hematocrit adjustment. The strongest associations, including resistin–IL-6 and R/A–IL-6, changed minimally, whereas several weaker associations were attenuated or shifted.
